# Surface imaging, laser positioning or volumetric imaging for breast cancer with nodal involvement treated by helical TomoTherapy

**DOI:** 10.1120/jacmp.v17i5.6041

**Published:** 2016-09-08

**Authors:** Frederik Crop, David Pasquier, Amandine Baczkiewic, Julie Doré, Lena Bequet, Emeline Steux, Anne Gadroy, Jacqueline Bouillon, Clement Florence, Laurence Muszynski, Mathilde Lacour, Eric Lartigau

**Affiliations:** ^1^ Medical Physics, Centre Oscar Lambret and Université Lille 1 Lille France; ^2^ Academic Radiotherapy Department Centre Oscar Lambret and Université Lille 2 Lille France

**Keywords:** breast cancer, radiotherapy, image‐guided radiotherapy, tomotherapy

## Abstract

A surface imaging system, Catalyst (C‐Rad), was compared with laser‐based positioning and daily mega voltage computed tomography (MVCT) setup for breast patients with nodal involvement treated by helical TomoTherapy. Catalyst‐based positioning performed better than laser‐based positioning. The respective modalities resulted in a standard deviation (SD), 68% confidence interval (CI) of positioning of left–right, craniocaudal, anterior–posterior, roll: 2.4 mm, 2.7 mm, 2.4 mm, 0.9° for Catalyst positioning, and 6.1 mm, 3.8 mm, 4.9 mm, 1.1° for laser‐based positioning, respectively. MVCT‐based precision is a combination of the interoperator variability for MVCT fusion and the patient movement during the time it takes for MVCT and fusion. The MVCT fusion interoperator variability for breast patients was evaluated at one SD left–right, craniocaudal, ant–post, roll as: 1.4 mm, 1.8 mm, 1.3 mm, 1.0°.

There was no statistically significant difference between the automatic MVCT registration result and the manual adjustment; the automatic fusion results were within the 95% CI of the mean result of 10 users, except for one specific case where the patient was positioned with large yaw. We found that users add variability to the roll correction as the automatic registration was more consistent.

The patient position uncertainty confidence interval was evaluated as 1.9 mm, 2.2 mm, 1.6 mm, 0.9° after 4 min, and 2.3 mm, 2.8 mm, 2.2 mm, 1° after 10 min. The combination of this patient movement with MVCT fusion interoperator variability results in total standard deviations of patient position when treatment starts 4 or 10 min after initial positioning of, respectively: 2.3 mm, 2.8 mm, 2.0 mm, 1.3° and 2.7 mm, 3.3 mm, 2.6 mm, 1.4°.

Surface based positioning arrives at the same precision when taking into account the time required for MVCT imaging and fusion. These results can be used on a patient‐per‐patient basis to decide which positioning system performs the best after the first 5 fractions and when daily MVCT can be omitted. Ideally, real‐time monitoring is required to reduce important intrafraction movement.

PACS number(s): 87.53.Jw, 87.53.Kn, 87.56.Da, 87.63.L‐, 81.70.Tx

## I. INTRODUCTION

TomoTherapy (Accuray, Sunnyvale, CA) breast treatments can be performed using the TomoDirect or Helical mode.^1^, ^2^ When internal mammary chain and clavicular nodes are involved, helical TomoTherapy is indicated and results in better coverage and better organ sparing compared to classical techniques, but it can also lead to larger low‐dose regions.^3^, ^4^

Several groups studied the precision of surface imaging systems for breast‐only or accelerated partial breast irradiation (APBI)^(5)^ treatments with the AlignRT (VisionRT, London, UK) and Catalyst (C‐Rad, Uppsala, Sweden) systems: APBI with portal imaging,^(6)^ whole breast with portal imaging,^7^, ^8^ APBI and orthogonal kV imaging,^9^, ^10^ APBI and CT‐on‐rails,^(11)^ phantom studies and patient tests,^12^, ^13^ and different patient localizations.^(14)^ Phantom studies, using specific couch displacements with phantom already in place, indicated mean total errors of less than 0.5 mm for VisionRT^(6)^ and less than 0.7 mm for Catalyst.^12^, ^13^, ^14^, ^15^, ^16^ The same type of study on patients and volunteers lead to mean total errors of 1.6 mm for visionRT and 1.1 mm for Catalyst. These results indicate the intrinsic precision, but not the day‐to‐day precision of patient positioning. Positioning of breast patients with nodal involvement on TomoTherapy adds two difficulties. Nodal involvement implies larger and more nonsurface volumes, with possible deformations due to arm or chin displacement. Also, TomoTherapy positioning is performed outside the gantry in the virtual isocenter, but treatment is performed inside the gantry, leading to a slightly different position due to couch flex, depending on patient weight and treatment localization. Couch flex is compensated for by mega voltage computed tomography (MVCT) imaging as the MVCT is performed in the real isocenter, but not for external‐based positioning.

When positioning patients based on lasers, volume deformations due to an incorrect arm or chin position can be present. If these are seen after taking a MVCT image, a compromise could be accepted or the patient can be repositioned, requiring a new MVCT. An initial incorrect yaw or pitch for the patient's position results in the same issue; TomoTherapy cannot currently compensate for these.^(17)^

The main goal of the introduction of an optical surface imaging system for TomoTherapy was to have first‐time‐right positioning^(13)^ and the possibility to reduce MVCT imaging, and thus also reduce dose (1–3 cGy/MVCT).^(18)^. We want to compare three modalities: laser‐based, surface‐based, and MVCT positioning. In order to compare laser‐based and surface‐based positioning, we can use the MVCT fusion results. The total MVCT positioning precision, in the absence of real‐time tracking,^(19)^ is a combination of intrafraction movement during the MVCT and image fusion process and the interoperator variability of MVCT fusion.

## II. MATERIALS AND METHODS

### A. Patient positioning procedure and treatment planning

Patients with breast cancer involving internal mammary chain and clavicular nodes are routinely treated with TomoTherapy in our center. Positioning is started with aligning the patient in the TomoTherapy virtual isocenter using lasers or Catalyst. The couch is displaced 70 cm towards the real isocenter in the cranial direction (+Y) and a daily MVCT image is taken. We use coarse slices and fine reconstruction leading to 3 mm slice thickness. After this MVCT image, the couch is displaced back to the virtual isocenter, after which the MVCT image fusion with the planning kVCT is performed. Finally, the position is corrected for in X, Y, Z and roll (left–right, craniocaudal, ant–post, roll) and displaced back towards the gantry for treatment.

All treatments were performed in 25 fractions. Boost volumes, when present, were treated by delivering 2.4 Gy/fraction as integrated boost. Treatment planning is performed in the helical mode with TomoEdge^(20)^ (Accuray) using a 5 cm field width and using details described in the previous work by Crop et al.^(21)^ This results in a treatment beam‐on time of approximately 5–7 min. Typical volumes of the different PTVs were in the order of 40 cm^3^ for internal mammary chain PTV, 1000 cm^3^ for the breast, 100 cm^3^ for infraclavicular node, and 100 cm^3^ for the supraclavicular node.

The patient is positioned with both arms up on a breast board with an inclination of 7°. The TomoTherapy couch is positioned as low as possible: 21–22 cm below the virtual isocenter. This allows for the highest amount of liberty, low thread effect,^(22)^ and better Catalyst camera view. The difference between the initial position and positioning result after MVCT fusion was evaluated in order to compare laser‐based and Catalyst‐based positioning. Forty patients with Catalyst‐based setup and 55 patients with laser‐based setup where included, resulting in, respectively, 810 Catalyst‐only sessions and 666 laser positioning‐only sessions. Thirty‐one of these patients had both laser‐based and Catalyst‐based setup on different days. Couch sag for TomoTherapy is different in comparison with the initial kVCT image. This leads to a different height position of the patient in the real isocenter, 70 cm further located, compared to the position in the virtual isocenter. This couch sag thus depends on the patient weight and the treatment location. Couch sag was evaluated with two methods: by adding weight to a MVCT‐scanned phantom and as the uncorrected mean Catalyst‐image bias between patient position outside the bore and inside the bore.

### B. Laser‐based positioning

For TomoTherapy, there are two sets of lasers: red lasers and green lasers.^(23)^ The green lasers are fixed and correspond to the virtual isocenter, 70 cm in caudal direction (Y) from the real machine isocenter. The red lasers are mobile and are used to indicate the setup reference point, relative to the virtual isocenter. Laser‐based positioning was performed using two sets of three points on the patient. The first set corresponding to the TomoTherapy red lasers is used for positioning, based on the reference point marked on the patient. The second point set is used for pitch and yaw evaluation, using both red and green lasers.

### C. Catalyst‐based positioning

**Figure 1:**
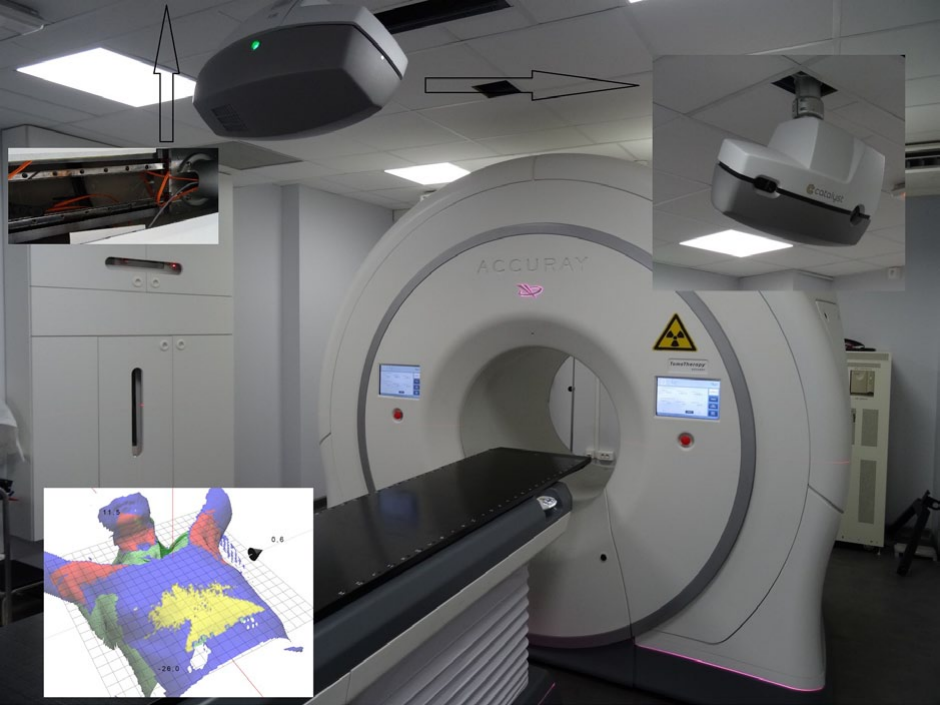
Catalyst camera in the TomoTherapy room. The camera is attached to a rail on the ceiling to facilitate maintenance of the TomoTherapy machine. Lower left inset: view of the live (blue) and reference (green) contours. The red and yellow zones are also projected live on the patient, representing regions that are too much anterior (red) or posterior (yellow). This example shows a pitch issue with the patient and the breast board.

The Catalyst system^(15)^ was introduced in 2014 for breast patient positioning for TomoTherapy in our center (Fig. 1). The Catalyst system acquires a structured light image^(24)^ of the patient's surface. This real‐time surface scan can be compared to a) the external surface of the initial kVCT, not requiring an additional camera, or b) a Catalyst reference at kVCT which requires a camera at kVCT location, or c) a Catalyst reference obtained at the treatment machine. The use of the external surface of the kVCT was compared with the use of a new reference at treatment machine by comparing the standard deviation of the final position result from MVCT imaging.

Catalyst applies a deformable registration based on a graph‐based nonrigid ICP algorithm^24^, ^25^ between the on‐line patient surface image and a reference. This algorithm provides a six degrees of freedom positioning result with the center of the planning target volume (PTV) as pivot point. The PTV gravitational point is recommended for TomoTherapy as the virtual isocenter is positioned inside the patient and not in the breast gland. The imaging region is determined by a box, but the surface is also weighted depending on distance from the PTV gravitational point: structures far away have low influence, even when in the image. Breathing effects are reduced by limiting the box just below the breast region and by applying a running average image of 6 s. Next to the six degree positioning, there is also a visible color map projection on the patient's surface to guide the technologists for the position (inset Fig. 1). Red color indicates that a region is too high (anterior) and yellow equals too low (posterior), compared with the reference image. The combination provides direct visual information projected on the patient for rotations and displacements (lower‐left inset Fig. 1). For Catalyst‐based positioning, lasers are only used for breast board alignment in the Y direction, by using the integrated ruler on the breast board.

The dicom RTPlan and RTStruct are imported into the Catalyst software after treatment planning. The kVCT‐based external contour from the RTStruct is used as the initial reference. Two parameters, gain and integration time, can be adjusted. These are camera specific: we used 7000 μs integration time and 400% gain as standard values. These have to be adjusted slightly for patients with extreme pale or dark skin due to the difference in reflection. Finally, the camera is attached to a rail such that the camera can be displaced easily and the TomoTherapy covers can be removed during maintenance (inset of Fig. 1).

Difference in standard deviation of the positioning precision between Catalyst‐ and laser‐based positioning was evaluated by using the modified robust Brown‐Forsythe Levene‐type (BFL) test, lawstat package^(26)^ of the R project,^(27)^ on the basis of the absolute deviations from the median. This test is also robust towards nonnormality of the data.

### D. MVCT positioning precision

MVCT positioning is always performed after laser‐based or Catalyst‐based positioning. The total MVCT precision can be assessed by evaluating the combination of both interoperator variability and the uncertainty associated with the movement of the patient during the MVCT and fusion process, which can take more than 4 min before treatment starts.

#### D.1 MVCT fusion interoperator variability

The baseline MVCT fusion result is susceptible to variability as each user gives a slightly different result. The results of the total interoperator variability of the MVCT fusion indicate the precision limit: laser positioning or Catalyst positioning cannot perform better than this standard deviation.

Three patients were selected, and for each patient, four sessions were selected. These four sessions consisted of two sessions using Catalyst‐based positioning and two sessions where only laser‐based positioning was used. Ten users, nine technologists and the attending MD, were requested to perform the 12 MVCT fusions twice: once by starting with the automatic fusion result and once by not using the automatic fusion result. This was a blind test: the 10 users did not know if the patient was positioned with or without Catalyst. The X, Y, Z positioning and roll were evaluated; yaw and pitch cannot be adjusted using TomoTherapy. The × and Z values obtained were corrected for the roll in the analysis; when there is roll present, the × and Z positions are affected. The correction is based on a mean distance and angle from the rotation center per patient and is dependent on the left‐ or right‐sided breast. However, this correction was small because the rotation angle is often small.

Using the same dataset, we tested if the different users gave more consistent results when the patient was positioned using Catalyst or not. Differences in standard deviations were evaluated using a BFL test (see Materials and Methods section C above).

#### D.2 Performance of the TomoTherapy MVCT automatic fusion algorithm

The automatic fusion result was compared with the median results of all users on a treatment session‐per‐session basis at a 95% CI level.

#### D.3 Intrafraction movement

Another important factor in the evaluation of the precision of MVCT precision is the intrafraction movement. As the MVCT image and fusion can take 4 min or more, the patient can have moved before treatment. This time factor must be included, in the absence of real‐time tracking, when evaluating total precision for MVCT positioning. Intrafraction movement was measured for 292 treatment sessions by obtaining additional snapshots using the Catalyst system, before MVCT, after MVCT, and after treatment. Eventual table displacements were subtracted from these position results. Data for two volunteers were added also.

## III. RESULTS

### A. Patient positioning procedure

A mean of 5 mm couch sag was found for breast patients. This mean couch flex was added during import for the Catalyst reference, based on the external patient CT contour, in the virtual isocenter.

### B. Laser‐based precision

The results of the deviations observed for laser‐based positioning are represented in red in 2, 3 (cumulative absolute histogram). The graphs represent 666 laser based sessions. The final results are represented in Table 1. For our patients, we obtained a standard deviation (68% CI) of 6.1 mm, 3.8 mm, 4.9 mm, and 1.1° for laser‐based positioning.

**Figure 2:**
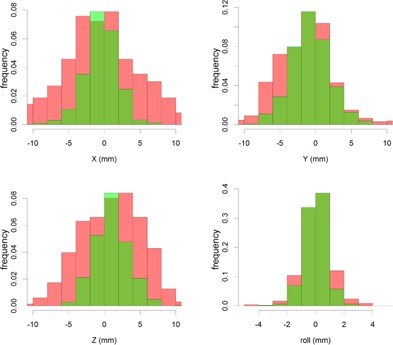
Histogram of laser (red)‐based positioning vs. Catalyst (green)‐based positioning. The laser‐based Z distribution has been corrected for the mean couch flex for fair comparison.

**Figure 3:**
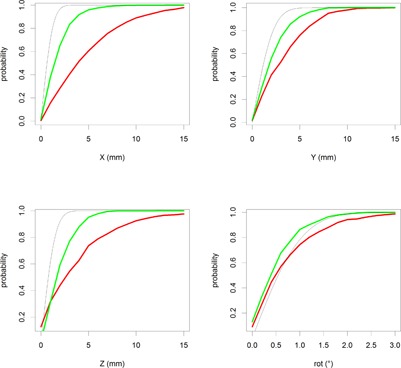
Graphs representing the probability of positioning the patient within specific absolute distance for Catalyst‐based (green, 810 sessions) and laser‐based positioning (red, 666 sessions) of breast patients. The black line represents the theoretical maximum (interoperator variability of MVCT fusion positioning).

**Table 1:** Summary of the standard deviations for laser‐based, Catalyst‐based, and MVCT‐based positioning. The p‐value represents the result of the BFL test between the respective categories above.

	*SD (X) (mm)*	*SD (Y) (mm)*	*SD (Z) (mm)*	*SD (roll) (°)*	*Nr*
Lasers	6.1	3.8	4.9	1.1	666
Catalyst	2.4	2.7	2.4	0.9	810
p‐value	<0.001	<0.001	<0.001	<0.001	
Catalyst CT ref	2.3	2.7	2.3	0.6	568
Catalyst new ref	2.5	2.8	2.4	0.9	242
p‐value	0.04	0.4	0.99	<0.001	
Patient motion					
4 min	1.9	2.2	1.6	0.9	292
10 min	2.3	2.8	2.2	1.0	292
MVCT interuser var	1.4	1.8	1.3	1.0	190
Total MVCT precision					
4 min before trt	2.3	2.8	2.0	1.3	
10 min before trt	2.7	3.3	2.6	1.4	

### C. Catalyst‐based precision

The respective results for Catalyst‐based positioning are shown in green in 2, 3. These represent 810 Catalyst sessions and result in a standard deviation (68% CI) of 2.4 mm, 2.7 mm, 2.4 mm, and 0.9° for Catalyst‐based positioning. BFL tests indicate a statistical significant difference with laser‐based positioning (p‐values <0.001, see Table 1). The eventual use of the a new reference in the treatment room, compared with the external kVCT‐based contour, did not result in significant different results (see Table 1).

The reproducibility of the camera position was evaluated using the daily QA phantom. The 238 instances over a year's period resulted in a mean of X/Y/Z (−0.002 mm,−0.006 mm, 0.014 mm) with a SD of (0.34 mm, 0.34 mm, 0.5 mm). A Student's *t*‐test could not indicate a statistically significant difference when having used the rail for maintenance.

### D. MVCT positioning precision

#### D.1 MVCT fusion interoperator variability

The dataset contains 190 data points from the theoretical 240 data points. Not all users completed the full dataset, leading to 47 missing values, and there were three extreme illogic outliers/errors. However, the dataset is still balanced for evaluation: blanks and outliers were distributed evenly over the different studied categories (see Table 2).

The interoperator variability tends to be lower when starting with the automatic fusion result, but the difference was not statistically significant (p‐value >0.05, see Table 2). Interoperator MVCT fusion variability for breast with nodal involvement, after initial Catalyst positioning, seems lower, but could not be validated by the BFL test (p‐values >0.05, see Table 2). Therefore, the interoperator variability for the 190 manual fusion results was evaluated as 1 SD, 68% CI (X, Y, Z, roll): 1.4 mm, 1.8 mm, 1.3 mm, and 1°, respectively.

The Y direction interoperator variability was larger than the variability in the × and Z directions. The p‐values of the BFL test were, respectively, <0.001 and 0.004.

**Table 2:** Interoperator variability of MVCT fusion process (standard deviation, expressed in mm) when starting with (auto) or without the fusion process from the automatic TomoTherapy fusion result (no auto). The p‐values represent the statistical significance of the Brown‐Forsythe Levine test. The subsequent diagonal p‐values represent this comparison.

		*Catalyst*	*Laser*	*p‐values*
Auto	SD(X)	1.0 mm^a^	1.5 mm	0.12
	SD(Y)	1.7 mm^a^	1.8 mm	0.89
	SD(Z)	1.2 mm^a^	1.2 mm	0.80
	SD(roll)	0.8°^a^	1.1°	0.19
	nr	53^a^	43	
No auto	SD(X)	1.5 mm	1.5 mm^a^	0.51
	SD(Y)	1.7 mm	2.0 mm^a^	0.64
	SD(Z)	1.2 mm	1.4 mm^a^	0.39
	SD(roll)	1.0°	1.2°^a^	0.48
	nr	52	42^a^	
p‐values	SD(X)	0.04	0.41	0.00^a^
	SD(Y)	0.66	0.55	0.42^a^
	SD(Z)	0.85	0.65	0.46^a^
	Roll	0.16	0.58	0.05^a^

^a^ The diagonal comparison (Catalyst, auto vs. laser, no auto).

#### D.2 Performance of the TomoTherapy MVCT automatic fusion algorithm

**Figure 4:**
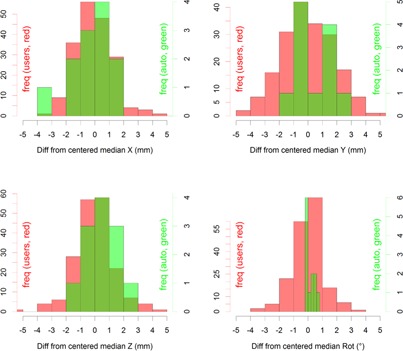
MVCT fusion results showing differences of the user‐dependent MVCT fusion result with the median result of each patient session. The results of all users (190) are shown in red; the difference between the initial automatic fusion result and this median is shown in green.^(12)^

The differences of the user fusion results (190) with the median of all users for each specific MVCT image are represented in Fig. 4 in red. The differences between the automatic fusion result and the median user result are represented in green.

No statistically significant difference was found between the user‐adjusted fusion results and the original result given by the automatic MVCT fusion algorithm without manual intervention. Specifically, the roll correction was found to have less variability for the automatic fusion compared to the user's manual correction results.

#### D.3 Intrafraction movement

**Figure 5:**
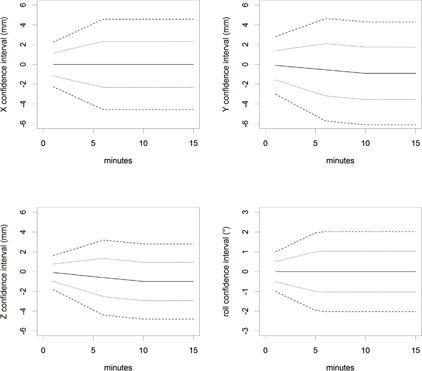
Intrafraction movement model (random and systematic patient movements combined). 95% CI = dashed, 68% = dotted, drift = solid line. There is a small drift in the Y and Z directions (patients slightly sliding down). The random movements increased with time (SDs increasing in time).

The intrafraction confidence intervals combining drift and irregular movements of the patients are shown in Fig. 5. We obtained total SDs (68% CI) of a) after 4 min: (1.9 mm; 2.2 mm; 1.6 mm; 0.9°), and b) after 10 min: (2.3 mm; 2.8 mm; 2.2 mm; 1°). The mean total root mean square (RMS) for all patients was evaluated as 3.3 mm with a SD of 2 mm after 4 min, and a mean total RMS of 3.8 mm with a SD of 2.4 mm after 10 min. The SD results (Table 1) should be combined with the interoperator variability of the MVCT fusion in order to obtain total MVCT precision. This results in SDs of a) after 4 min: (2.3 mm; 2.8 mm; 2.0 mm; 1.3°), and b) after 10 min: (2.7 mm; 3.3 mm; 2.6 mm; 1.4°). The largest increase in random position happens in the first 5 min time span.

## IV. DISCUSSION

We compare laser‐based positioning with surface‐based positioning by using the MVCT fusion results. The issue with this comparison is the lack of exact truth to compare with. In an APBI study,^(10)^ surgical clips were used as reference in order to calculate the registration error. In the present study we consider breast with nodal involvement, and cannot apply this methodology. Other studies focusing on APBI or breast‐only^6^, ^7^, ^9^, ^10^, ^11^ consider more limited surface and volume. Moreover, most of these studies use the user‐defined portal image position as exact position. When treating the MVCT or portal image as “reality”, one encounters an issue with interoperator variability and patient movement. As a real example, when performing a manual fusion between original kilovoltage CT (kVCT) and MVCT, one person will indicate a (X/Y/Z/roll) (−6 mm,−1 mm, 0 mm, 0°) correction, whilst another user indicates (−4.2 mm,−4.1 mm,−1.3 mm, 0.8°) and yet another user (−3 mm,−3 mm, 0 mm, 1°). Even the same user will never indicate the same result twice: for example (−6 mm,−1 mm, 0 mm, 0°) the first time, whilst a second time (−3.4 mm,−1.2 mm, 1.1 mm, 0.5°). When evaluating MVCT positioning with an auxiliary positioning system, laser‐ or surface‐based, one needs to take into account the inherent interoperator variability of the MVCT fusion result.

### A. Catalyst‐based positioning

Catalyst‐based positioning performed statistically significant better than laser‐based positioning. The uncertainties associated are very close to MVCT imaging. Important to note is that the results for Catalyst‐based and laser‐based positioning include both MVCT interoperator variability and partial intrafraction movements: the time between positioning and MVCT

(1–3 min). When edema begins to appear after several fractions, it is expected that surface positioning will behave differently,^(11)^ but this was not explored in detail in the current study. Catalyst also shows a real‐time image. This offers the possibility to verify if patients breathe correctly through the diaphragm and do not present large chest wall breathing.

Tomotherapy couch flex, which depends on patient weight and treatment location, can introduce a bias for the initial kVCT‐based reference surface image. We corrected this at import by adding 5 mm in the Z direction for breast patients.

### B. MVCT positioning precision

#### B.1 Interoperator variability

We observed that the interoperator variability in the Y direction was higher compared to the other directions. This was most likely due to the TomoTherapy MVCT Y direction resolution as coarse slices with 3 mm thickness were used, compared to 0.78 mm/pixel in the X/Z direction.

Our tests for MVCT interopterator variability indicate that Catalyst‐based positioning, followed by MVCT, automatic fusion and manual correction, resulted in the lowest variability, but this was only statistically sound in the × direction and roll. Therefore we could make no conclusions in the difference in MVCT interoperator variability between originally laser‐based or Catalyst‐based positioning, nor in the difference between using the automatic fusion process or not using it.

#### B.2 Performance of the TomoTherapy MVCT automatic fusion algorithm

Our results indicate that the fusion process should involve verification and, at most, a correction for a gross error instead of adjusting the images on a submillimeter basis. The interoperator variability was larger, and the automatic algorithm was more consistent for breast patients. For roll corrections this was even more important: user corrections actually add variability.

#### B.3 Intrafraction movement

The random movements were slightly higher compared to Wiant et al.^(19)^ This could be due to a) the lower temperature in the treatment room as the ambient temperature for TomoTherapy is 20°C due to the air cooling, b) discomfort of the breast board used, or c) the act of the couch moving in and out of the bore for MVCT and treatment, influencing the patient position.

A mean shift of 1 mm in the Y and Z positions was found after 10 min and was most likely linked with patients sliding slightly from the breast board and/or patients relaxing during the treatment. Wiant et al.^(19)^ obtained a mean shift of 0.44 mm. This difference is probably also part of the reason why the mean RMS of all patients was slightly higher in our results: 3.8 mm compared to 2.98 mm in the Wiant study after 10 min.

### C. Laser vs. Catalyst vs. MVCT precision

We see that Catalyst performs about equally as MVCT positioning, when taking into account the time between initial positioning and start of treatment. We wish to point out that the Catalyst and laser positioning results are based on the MVCT fusion afterwards. This incorporates thus the interoperator variability of the MVCT fusion and a small movement possible between positioning and start of MVCT (±1 min).

Comparison with other surface positioning studies is difficult as most other studies consider APBI or breast only studies, or compare to portal images/orthogonal images with inherent interoperator variability. Bert et al.^(6)^ (APBI/portal) found total SDs of 4.4 mm for lasers and 4.2 mm for portal images. Shah et al.^(7)^ (whole breast) found mean displacement differences between the surface based positioning and portal imaging, but also a random error of (2.2 mm; 3.2 mm; 2.2 mm), totaling 4.5 mm. We obtained a slightly lower 4.3 mm SD of the total vector, but for breast with nodal involvement. In the case of breast with nodal involvement, there is no “ground truth” to compare with, as done by Chang et al.^(9)^ using surgical clips.

The roll corrections appear to be equal between Catalyst‐ and laser‐based positioning. The underlying reason is shown in Fig. 3(d): the roll corrections are equal to the MVCT interoperator variability. This finding indicates that the automatic fusion correction result should be trusted and not adjusted. This is consistent with the rigid phantom results of Laub et al.^(28)^

These results can be used on a patient‐per‐patient basis to decide which positioning system performs the best. If the standard deviation of the MVCT fusion result of the first five fractions is below, which, after 4 and 10 min, is (2.3 mm; 2.8 mm; 2.0 mm; 1.3°) and (2.7 mm; 3.3 mm; 2.6 mm; 1.4°), then it is theoretically better to not use a MVCT scan at all, but to rely on Catalyst‐only positioning and treat immediately. However, depending on the treatment beam‐on time, the position uncertainty will be higher again near the end of the treatment fraction, corresponding to the inferior region for TomoTherapy.

## V. CONCLUSIONS

Catalyst‐based positioning was found to be more precise than laser‐based positioning. Depending on the patient and in the absence of real‐time monitoring, Catalyst positioning can perform better than MVCT imaging due to the immediate treatment after positioning and thus reducing patient movement before treatment starts. The stability of patients on the breast board is thus of major importance.

Interoperator MVCT fusion variability plays an important role in the analysis of positioning results. An example is the user's introduction of larger variability for roll correction compared to the automatic setup. When combining surface‐based positioning with MVCT imaging, automatic fusion performs very well for breast patients, and manual adjustments should be limited, especially for roll corrections.

## ACKNOWLEDGMENTS

We wish to thank the reviewers for their invested time and constructive comments, which have improved our manuscript substantially.

## COPYRIGHT

This work is licensed under a Creative Commons Attribution 3.0 Unported License.

## Supporting information

Supplementary Material

Supplementary Material
