# Supplementary material for: Surface imaging, laser positioning or volumetric imaging for breast cancer with nodal involvement treated by helical TomoTherapy
Source: J Appl Clin Med Phys. 2016 Sep 8;17(5):200–11. doi: 10.1120/jacmp.v17i5.6041 (PMC5874112; doi:10.1120/jacmp.v17i5.6041)
Supplement: Supplementary file 1 — Supplementary Material [file ACM2-17-200-s001.docx]

Surface imaging or laser positioning for breast cancer helical TomoTherapy

**F. Crop (1), D. Pasquier (2), A. Baczkiewic (2), J. Doré (2), L. Bequet (2), E. Steux (2), A. Gadroy (2), J. Bouillon (2) and E. Lartigau (2)**

1. Medical Physics, Centre Oscar Lambret, *3, Rue Frédéric Combemale, 59000 Lille, France*
2. Academic Radiotherapy department, Université de Lille, Centre Oscar Lambret, *3, Rue Frédéric Combemale, 59000 Lille, France*

**Running title:** MVCT and Catalyst precision
