# Supplementary figures and images for: Surface imaging, laser positioning or volumetric imaging for breast cancer with nodal involvement treated by helical TomoTherapy
Source: J Appl Clin Med Phys. 2016 Sep 8;17(5):200–11. doi: 10.1120/jacmp.v17i5.6041 (PMC5874112; doi:10.1120/jacmp.v17i5.6041)

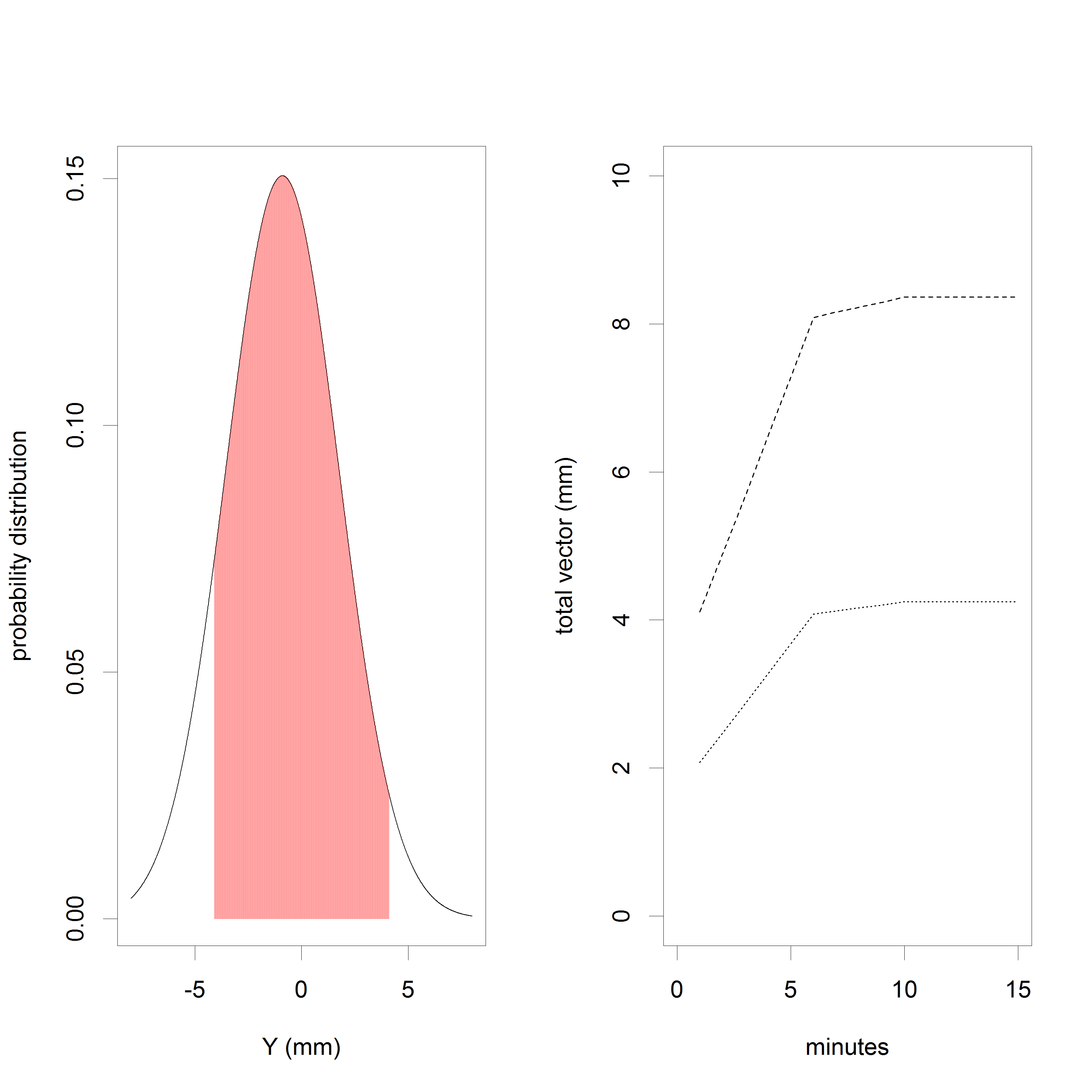

Supplement: Supplementary file 2 — Supplementary Material [file ACM2-17-200-s002.png]
